# Its History and Development: The Missions' Share

**Published:** 1921-02-26

**Authors:** 


### MEDICAL EDUCATION IN CHINA.

### Its History and Development: the Missions' Share.

SPEECHES and an address of absorbing interest rewarded the gathering that assembled in the Barnes Hall of the Royal Society of Medicine on Monday, February 21. The audience were members of the medical profession, and the meeting was convened for the purpose of making known the progress and the prospects of medical education in China. Sir Donald MacAlister, K.C.B., was in the chair, and he was supported on the platform by men distinguished in various branches of the profession, by Mr. O. King representing the Chinese Ambassador and by the Right Hon. Sir John Jordan, G.C.M.G., whose name is well known to all students of Chinese history and administration.

Sir DONALD MACALISTER, in welcoming the audience, stated his conviction that in a land of ancient and conservative culture like China, with its venerable philosophy of practical life, Christianity is called upon to demonstrate by its actual works, as well as by its faith, that it has a better and more beneficent way to offer as well for the body as for the soul. Christianity must practise the Founder's method, as well as preach His message, if it is to impress the very practical mind of China. But when once the first impression is made and the seeds of Christianity are wisely planted in Chinese soil, its future spread and growth will be conditioned by the measure in which it ceases to be regarded as foreign and is adapted and adopted as Chinese. To become most widely diffused it must be mediated to the people not by foreign Christians, however devoted, but by Chinese Christians. Foreign Missions of Western civilisations have their indispensable place and work, and will for a generation have a vast field to cultivate. But their conscious and avowed aim ought to be not merely to plant colonies, but to prepare Chinese Christians for the founding of a native and self-propagating Church of China; not merely to establish foreign doctors in Chinese hospitals, but by education to build up a native Chinese medical profession capable of making its own contribution to the international stock of medical knowledge. The progress of a live medical mission should be estimated, not by the number of its patients, but by the number of its native students and graduates.

Dr. HAROLD BALME, Dean of the School of Medicine of the Shantung Christian University, Tsinan, then read an illuminating paper on "The Advance of Medical Education in China." He shortly traced the introduction of Western medicine into China, starting from the surgeon of the East India Company, Thomas Colledge, who in 1827 opened a voluntary hospital there. He was followed by the first medical missionary to China, an able surgeon from the United States, Peter Parker by name, whose fame was so extraordinary that patients waited in the streets overnight in order to be in time to secure an out-patient ticket in the morning. The Gordon Museum of Guy's Hospital contains a series of paintings by a celebrated Chinese artist portraying surgical conditions which Parker had cured by his wonderful skill in operation—all these in pre-anæsthetic days, be it remembered. Colledge and Parker saw that China's needs would never be met except by the trained efforts of her own people, and they set to work to train Chinese students in scientific medicine. Such training could only consist of oral instruction, as there were no Chinese text-books nor any facilities for laboratory work. They started with training three men, of whom one, Dr. Kwan-tao, later obtained a great reputation as a surgeon. During the next fifty years hospitals sprang up in various parts of the country, almost always in connection with the work of medical missions; training classes were organised and medical text-books translated into Chinese, but it was not till 1881 that the first Chinese medical college was established. Its establishment was due to the joint efforts of a medical missionary, Kenneth Mackenzie, and the great Viceroy Li Hung Chang. This was the college at Tientsin, and though at first its course was rudimentary, it laid the foundations for the later remarkable advance in medical education in China. The Hong Kong College was the next to be established, and to it Sir Patrick Manson and Sir James Cantlie were attached as respectively Dean and Professor of Anatomy and Surgery. Other small medical schools came into existence, one being for women students only, founded as far back as 1899 by Dr. Mary Fulton in Canton. The first medical college to be established in China by the co-operation of both British and American medical men and of missionary societies representing different religious denominations was the Peking Union Medical College, which was organised by Dr. Thomas Cochrane after the Boxer uprising of 1900. It was recognised by the Chinese Government from the outset, and the Board of Education gave a special diploma to all graduates and made an annual grant towards its upkeep. Many other missionary medical colleges sprang up, and in 1913 the China Medical Missionary Association passed a resolution urging that no further medical colleges be established until those in existence—eight for men and three for women students—were made efficient.

The influence of Chinese medical men who had been trained on modern lines, mostly in Europe and America, was then beginning to be felt, and the Government was urged to establish modern standards of medical education. Other organisations, too, pressed forward the cause of medical education. The Harvard Medical School at Shanghai was established, and the Japanese Medical College at Moukden, as well as two German medical colleges since closed. But the greatest contribution to the cause of medical education in China has been effected by the Rockefeller Foundation of New York, which sent out two influential Commissions in 1914 and 1915. They entered into negotiations with the London Missionary Society, and have taken over the complete financial responsibility of the Peking Union Medical College. The language employed for teaching is English, and this led to a desire for at least one medical school teaching in Chinese. The China Medical Missionary Association, through its Council of Medical Education, took the matter in hand, and urged that all missions interested in the advance of medical education, through the medium of Chinese, should concentrate their forces in the development and support of such a school. To effect this the colleges at Nanking and Hankow were given up, and their students and certain members of the staffs were transferred. The School of Medicine of the Shantung University at Tsinan is the outcome of their efforts; the Rockefeller Foundation made a grant of £30,000 towards the cost of additional buildings and equipment, a grant which has since been extended. This Dr. Balme stated to be probably one of the most remarkable instances of missionary co-operation in China, for it brought no fewer than nine missionary societies of Great Britain, Canada, and the United States into co-operation. It has secured to the school a faculty of over twenty full-time teachers, representing some of the leading universities and hospitals of Great Britain and North America, and the maintenance of modern standards throughout. This stimulus is being felt in other parts of China. In Moukden Dr. Christie is at work, and in Chengtu, Shanghai, and Changsa similar efforts are being made. The Council of Medical Education has got ahead of England by requiring a two-year course of pre-medical instruction to be taken by students previous to entering upon their five-year medical course. To all that pertains to the development of medical education the Chinese Government extends the greatest sympathy and encouragement. Serious financial problems are, however, facing some of the newer medical schools.

Sir A. Pearce Gould proposed a resolution placing on record the sympathy of the meeting with the efforts of the Chinese Government and of British medical practitioners to co-operate in the establishment of medical schools of the highest standard, and its willingness to promote in every way possible the advance of medical education in China. He referred to the story of the advance as one unequalled in the history of the world. Mr. McAdam Eccles said he believes medical education in China to be the great link of co-operation between the peoples of Great Britain and her Dominions, the peoples of the United States, of the great country of China, and the missionary societies—a link which will diminish, or even abolish, the so-called Yellow Peril.

In reply to questions, Dr. Balme and Professor Earle (Hong Kong University) indicated ways in which the medical profession here can help otherwise than financially. Dr. Balme desires sympathy for the efforts of medical missionaries, acquiring a knowledge of their work, and recruiting men for various posts; as high a standard for professors in China is required as here—nothing less. Professor Earle hopes that help may be given by granting post-graduate facilities to Chinese graduates. Dr. Sambon, in entering a plea for the fuller study by our medical men of the medical writings and practice of the ancients, more particularly of China and the East, asked for hospitality for Chinese students here.

Mrs. Scharlieb, in proposing a vote of thanks to the Chairman, said that the advance of medical education in China was not a national, an Imperial, or a Western enterprise, but a great Christian enterprise bringing its own reward and blessing.

Sir John Jordan, G.C.M.G., who said he spoke as a layman, dwelt with emphasis on the value of medical missionaries; the medical missionary is the one person who can approach most closely to the Chinese in the present day. England has no realisation of the work that is being done by doctors in China; if it had it would not allow the Peking Hospital, established in 1860 and carried on for some years, to be passed over to the control and support of the Americans. In the co-operation of Great Britain and America the future not only of the Far East but of the whole world lies, and unless England does her share the prestige of this country in China will suffer. This country does not at present contribute anything like what America does either in men or money.

